# Experiences of community engagement for SARS-COV-2 and COVID-19 community surveillance in a rural and urban population in South Western Uganda

**DOI:** 10.4314/ahs.v24i3.3

**Published:** 2024-09

**Authors:** Dominic Bukenya, Bernard Mpairwe, Josephine Naluwugge Ssentongo, Gerald Ssenyomo, Julie Fox, Robert Newton, Janet Seeley, Joseph Okello Mugisha

**Affiliations:** 1 Medical Research Council/Uganda Virus Research Institute & London School of Hygiene and Tropical Medicine Uganda Research Unit, Entebbe, Uganda; 2 Global Health Institute, Kings College, London; 3 Health Sciences, University of York, York, UK; 4 Department of Global Health and Development, London School of Hygiene and Tropical Medicine, London, UK

**Keywords:** SARS-COV-2, COVID-19, community engagement, surveillance, acceptability, East Africa

## Abstract

**Background:**

We describe lessons learnt from community engagement activities for the successful implementation of a COVID-19 community surveillance study in Kalungu district, south-western Uganda.

**Methods:**

One rural and one urban site were selected for COVID-19/SARS-CoV-2 community surveillance in selected households and three public health facilities. COVID-19 pandemic and national COVID-19 protection measures were described and explained in meetings with: district, sub-county/town, village council officials, religious leaders, health workers and Community Advisory Board members. The community surveillance study was introduced to the meeting attendees and minutes captured in English/Luganda (subsequently translated to English). All minutes were manually coded and analysed thematically.

**Results:**

The minutes revealed that community members were generally supportive of the COVID-19 surveillance study. Members requested wider communities' sensitisation about COVID-19 and the survey beyond the selected households. Misinformation and mistrust of Government statements were widespread. People in the community did not understand the ‘random sample’ selection for the study. Providing appropriate medical care, face masks and honouring appointments for study participants and ensuring that COVID-19 prevention measures were followed during the study improved participation.

**Conclusion:**

Successful COVID-19 community surveillance required continuous, active community engagement between the research team, and community stakeholders while maximising previous gains and exploiting existing resources.

## Introduction

Community engagement is defined as the involvement and participation of individuals, groups and structures within a parameter of a social boundary or catchment area of a community for decision making, planning, design, governance and delivery of services^1^. Similarly, Johnston and Taylor^2^ defined, ‘community engagement’, as communication being based on a relational process that brings about understanding and evaluation, involvement, exchange of information and opinions about a particular issue or a project in a particular group of people (p.19). Effective community engagement is essential in addressing health emergencies^3^.

Recent Ebola and Zika outbreaks have shown that the simplest yet most effective method of organising and responding to health emergencies is to build trust and confidence between communities and those providing the services^4^, ^5^. Additionally, to work with communities safely, it is important to understand community views and to proactively share information^6^-^8^. Early implementation of Ebola prevention and control activities during the 2014-2015 outbreak in West Africa faced several barriers including suspicion about the existence of the associated disease and, the underlying motives of the governments and international organisations^6^,^9^,^10^. Community engagement became a key pillar to address these barriers^8^, making use of measures such as: building partnerships with local, political and religious leaders and working with the community members to develop and adjust key messages^6^, ^11^-^15^. Lessons which were once more rehearsed as the health authorities in Uganda tackled an Ebola outbreak in October 2022 16.

Community engagement has been critical for COVID-19 prevention, control and surveillance^7^, ^17^. Through a bottom-up approach, community members have been able to participate in decision making for the process of planning, design, governance, surveillance, case detection and contact tracing^8^, ^18^.

In Uganda, the first COVID-19 case was reported by the Ministry of Health on 20^th^ March 2020 (an international traveller from Dubai). Although the outbreak was initially in urban settings, there were growing fears over the spread of the pandemic in more rural and remote areas in the country^19^. Several research projects on COVID-19 have been undertaken in Uganda since the first case was identified, including work by the Medical Research Council/Uganda Virus Research Institute and London School of Hygiene and Tropical Medicine Uganda Research Unit (MRC/UVRI and LSHTM) Uganda Research Unit. The Unit initiated a research project to undertake surveillance for COVID-19 in Kalungu District, a place where community surveillance for HIV and other conditions has been in operation for over 30 years. The COVID-19 surveillance was put in place in one urban population (Lukaya) and in a rural population (Kyamulibwa sub-county). Lukaya town is located along the trans-African highway, frequently traversed by trucks travelling from Kenya to Rwanda, Burundi and Democratic Republic of Congo (DRC). It is also used by trucks entering Uganda from Tanzania, which was found to be the initial source of many SARS-CoV-2 infections. Kyamulibwa sub-county is the site of the General Population Cohort^20^.

The aim of this paper is to describe lessons learnt through the process of community engagement and how these lessons were important for the successful implementation of the COVID-19 community surveillance study. These lessons can inform policy, guidelines and standard operating procedures (SOPs) for future surveillance studies of other emerging infections.

### Study Setting

The SARS-COV-2 and COVID-19 community surveillance study was undertaken within the General Population Cohort (GPC) and in Lukaya town. The GPC is an open population cohort in Kyamulibwa sub-county and town council in Kalungu district, which is about 150 km SouthWest of Kampala. The GPC covers a population of about 22,000 people and was established in 1989 to study the epidemiology of HIV in a general population^20^. With time, the GPC- based studies have incorporated other diseases and conditions. Since early November 2020, studies on COVID-19 have been implemented within the GPC. The study population is spread across the countryside in neighbouring villages and Kyamulibwa town. Rain-fed agriculture is the main economic activity. The major ethnic groups are the Baganda (75%), immigrants from Rwanda (16%) and Burundi (3%) and other Ugandan and Tanzanian tribes (6%). Only 13% of the residents have attained education beyond primary level. The GPC studies are administered from the Kyamulibwa field station. Within each of 25 GPC study villages, there are individuals who record and report monthly vital events (birth, death, in migration and out migration). Participants of the GPC studies can obtain free medical care from the GPC clinic which is located at the Kyamulibwa field station next to the Kyamulibwa sub-county headquarters.

Lukaya town is about 90-100km SouthWest of Kampala along the trans-African highway and lies in between a rich agricultural hinterland to the west and several fishing villages along the shores of Lake Victoria to the east. The population of Lukaya town is approximately 25,000. The town is often a stop-over for long distance trucks from Kenya to western Uganda and the neighbouring countries, including Tanzania which was an initial source of SARS-CoV-2 infections in Uganda^19^. On average approximately 50-80 trucks make stop overs in Lukaya town for a period of between one hour to overnight stays. Along with the established shops, the town has numerous restaurants, hotels and bars catering for the passing traffic. Commercial sex is available in many of these venues. Furthermore, there is considerable mobility between Lukaya town, the agricultural hinterland and the neighbouring fishing villages. Lukaya town council is about 25 km from Kyamulibwa sub-county and town council and is linked to the GPC by feeder roads often used by bicycles, motorcycle taxis but rarely by other vehicles.

The government health care system operates through a tiered system. At the district level, there is a district health office overseen by an experienced medical doctor, charged with resource distribution including staff deployment to the district hospital and health centres IV, III and II. A health centre IV is run by a doctor and mandated to provide services at a constituency/county level and receives referrals from health centre IIIs, which are staffed by a clinical officer, nurses and midwives. The health centre II, which is run by an enrolled nurse, is the first contact of professional health care and these are either walk-ins or referrals from the ‘lowest’ tier, the village health teams. A village health team comprises of two village members in charge of promoting health in the village, especially primary health care. Diagnosis and management of uncomplicated chronic diseases including hypertension, diabetes, asthma and HIV are expected to be done at health centre IIs and IIIs. However, usually, health centre II teams refer suspected diabetic patients to health centre IIIs and higher-level facilities. There are health centres IV, III and II within the Kyamulibwa and Lukaya study settings^21^. There is a GPC clinic that serves participants from the study area. Only the health centre IIIs in both Lukaya and Kyamulibwa sub county and the GPC clinic were involved in the clinic-based part of the surveillance study.

The current study field activities were coordinated from the Kyamulibwa MRC Unit field station. This station hosts the General Population Cohort (GPC) described earlier. Within the GPC there are different departments. Among them are: mobilisation/community liaison/engagement, medical survey, clinic and data teams. The Social Science team at the station collaborates with the GPC team to pursue research questions that have behavioural and epidemiological components. In addition, the social science team nests studies pursuing social science-oriented research questions in the GPC. Community engagement activities precede any GPC study related activities in the community; the present study built on that experience. The authors of this paper are drawn from the GPC, community engagement and social science staff at the field station. All the authors working at the field station have extensive experience of both conducting research and living in the GPC area. The role of each author is detailed on page 9.

## Methods

The GPC SARS-CoV-2 and COVID-19 community surveillance study was a combination of two surveys namely a clinic and population-based household survey. The clinic survey was conducted at three main health facilities within the GPC and Lukaya. These included the GPC clinic, Kyamulibwa health centre III and Lukaya health centre III. Questionnaire data, blood samples and nasal swabs were taken from patients with unexplained fevers and other symptoms of COVID-19 (i.e. cough, influenza, headache, general malaise, being a contact). Samples were tested for SARS-CoV-2 through PCR testing at the Uganda Virus Research Institute (UVRI) Entebbe reference laboratory. Results were retrieved from the national COVID-19 dashboard and given to the participants 2-3 days after screening and, if positive, household contacts were also mobilised and tested. Participants who tested positive for SARS-CoV-2 from the clinic-based survey were followed up to provide the standard of care and treatment. For the household survey, participants from 500 households within the GPC and 500 households within Lukaya town were first surveyed for the household survey at baseline from November 2020 and followed up monthly for 18 months until April 2022. Everybody in the selected households irrespective of whether they had COVID-19 symptoms or not were eligible to enroll in the household survey. Similar COVID-19 screening and COVID-19 PCR swab data were collected and tested at the UVRI Entebbe reference laboratory. Results were retrieved in the same way as in the clinic-based survey. In addition, there was a qualitative methods component where data were collected using individual in-depth interviews and small group discussions/meetings to understand some deeper issues on the way the community perceived the pandemic and views on COVID-19 vaccines.

### GPC SARS-COV-2 and COVID-19 community surveillance study procedure

All study participants received information on the study and were asked for consent to participate. Those who agreed, signed/provided a thumb print on consent forms before taking part in interviews. Questionnaire data were obtained through face to face interviews from study participants at clinics or at home (depending on where they were recruited). The questionnaire covered demographic characteristics of study participants, symptoms and signs for COVID-19, pre-existing chronic conditions, alcohol consumption and tobacco use. After the interviews, anthropometric measurements were conducted. We also obtained 20 mls of blood at enrolment (10 mls SST and 10 mls EDTA) and 5 mls (EDTA) at monthly follow up visits. Point-of-care tests were used for haemoglobin measurement with a haemocue and rapid tests for HIV and malaria were performed following the Ugandan Ministry of Health Standard Operating Procedures for malaria and HIV rapid tests. After the point of care tests were done, the remaining blood and plasma was centrifuged, divided into cryovials and stored in liquid nitrogen at -196 degrees Celsius. The samples were later transported to Entebbe where there is a laboratory bio-repository unit for long term storage at -80 degrees Celsius.

The clinic-based survey which did not have a follow up component screened and enrolled 2542 participants of which 378 were diagnosed with COVID-19. The community household-based survey enrolled a total of 5241 participants, 298 of whom were at one time diagnosed with COVID-19 during the course of the study's 18 months' duration. The community household-based survey participants were followed up every month for a duration of 18 months. Health care was provided to all participants who tested positive for SARS-CoV-2 in the clinic and community household-based surveys according to the national guidelines.

### The qualitative methods component

Qualitative data were collected longitudinally from a randomly selected cross-section of 45 individuals aged 18 years and above who were diagnosed with COVID-19. The participants were selected from both the clinic and community household based surveys participants. Additional qualitative data were collected from nine small group meetings to explore households' experience of managing COVID-19 patients and the local response to non-pharmacological interventions. The small group meetings participants were selected from both the clinic and community household surveys. The average size of the small group meetings was three with a minimum of two and maximum of four participants. To avoid over-loading any one individual, efforts were made to ensure that no one participated in both household and individual qualitative data collection either interviews or small group discussions. This also allowed more people to have a chance to participate in the study.

### Community engagement approaches

Community engagement activities ahead of the GPC SARS-COV-2 and COVID-19 community surveillance study were rolled out to raise community awareness about COVID-19 and study plans. Leaders and community stakeholders at the district level and those in Kyamulibwa sub-county, Kyamulibwa and Lukaya town councils were categorised into nine different groups and invited for a community COVID-19 surveillance study introduction and familiarisation meeting. The leadership categories included: (i) Kalungu district leadership, (ii) Kalungu district health leadership including the COVID-19 task force, (iii) the Kyamulibwa sub-county, Kyamulibwa and Lukaya town local councils' leadership, (iv) religious leaders in Kyamulibwa sub-county, Kyamulibwa and Lukaya towns, (v) police and security leadership in Kyamulibwa sub-county, Kyamulibwa and Lukaya town council, (vi) village local council leadership in Kyamulibwa sub-county, Kyamulibwa and Lukaya town councils, (vii) health workers at the GPC clinic, Lukaya and Kyamulibwa health centre IIIs. (viii) GPC community advisory board, (ix) Village health teams (VHTs) and members of the households randomised to take part in the study. During these meetings, information on the global COVID-19 pandemic was given, national COVID-19 standard operating procedures were explained, and the COVID-19 community surveillance study was introduced, with a call to support the study uptake and advise on what needed to be done to improve its uptake. One meeting was held with each of the above community stakeholders. One general meeting that brought together the different community stakeholders described above was held at the launch of the GPC SARS-COV-2 and COVID-19 community surveillance study in early November 2020. In all ten meetings were held before the commencement of the GPC SARS-COV-2 and COVID-19 community surveillance study. In addition, the leaders in the study communities and at the district-level were invited at different times and briefed about the study, asked for their own views about COVID-19 given the changing national guidelines, and about the role of the COVID-19 vaccination programme. These meetings also explored what the community and district leaders' viewed as possible implementation challenges and how we could overcome them. They were asked to promote the study in the study areas, in addition to giving their communities information about COVID-19. A work plan was drawn up for the community engagement activities. The GPC SARS-COV-2 and COVID-19 community surveillance study coordinator or the community engagement/mobilisation team leader (second and fourth authors) facilitated the community engagement meetings. The third author took minutes for the community engagement meetings. The last, second, and fourth authors took turns to facilitate the general community engagement meeting at the study launch where minutes were taken by the third author. In all community engagement meetings, detailed minutes were captured.

Additional community engagement on COVID-19 awareness and study promotion was done via the media: radio, newspaper and television. Reporters from these media organisations were invited to cover the study launch. Thereafter, more community engagement, COVID-19 awareness and study promotion was done through a local community radio station. The community engagement and mobilisation team selected the radio station with the widest coverage/most listened too. The team was able to determine this because more than half of the community and mobilisation team members are resident to the GPC study area and have considerable community engagement and mobilisation working experience in the area. The resident study team members also helped to determine the community radio to use to cover the Lukaya town council. The second and fourth authors drafted the community engagement messages about the study that were broadcast over the community radio every weekend for a period of three months during the preparations for the surveys in the first year of the pandemic, when awareness raising about COVID-19 was critical.

### Community engagement data collection techniques

Data analysed for this paper were collected through the minutes of meetings and by documenting deliberations during the COVID-19 awareness sessions and community COVID-19 surveillance study briefings. Documenting the meeting deliberations involved; capturing what was presented about the study: why the study was being conducted, what samples and data were to be collected and study participants' selection/eligibility. Information was also shared on participants' time compensation and transport reimbursement, results handling and how those diagnosed with COVID-19 were to be handled. The community engagement meetings also asked the community stakeholders to support and promote the study uptake in their communities. Furthermore, the notes also captured meeting attendees' reactions to the information presented to them and action points agreed upon.

Meetings with district leadership were conducted in English while those with community leaders and the community advisory board were conducted in Luganda. Detailed minutes were taken either in Luganda or English depending on which language the meeting was conducted in. Meetings were not audio-recorded. Minutes were taken by JNS and GS who are experienced field workers. JNS translated the Luganda minutes into English while the GS and BM proof read the minutes to ensure completeness. BM would then print off a hard copy of the minutes for filing in the investigator' site file and also share a soft copy with study investigators.

### Data management and analysis

COVID-19 awareness and COVID-19 community surveillance study briefing sessions minutes conducted in Luganda were written up and translated into English. The minutes were proof read for accuracy and completeness. This constituted the first level of analysis. The second level of data analysis involved the first author (DB) reading all the minutes to become familiar with the data and also to identify emerging themes and patterns. The emerging patterns and themes were discussed and agreed upon by DB, JOM, JNS, GS and JS and these provided broad codes used in the data coding and analysis manually. Among the emerging themes were: leveraging existing resources, value/importance of information giving, community worries/fear and community based bottom-up suggested strategies. The third level of data analysis was thematic manual coding of the meeting minutes under the emerging themes and patterns. JNS coded the data with guidance from DB. These data were compared and contrasted with a view to preparing analytical memos on the key findings^22^,^23^. DB analysed the data manually and wrote the first draft with guidance from the JF, RN, JS and JOM.

## Results

We present the results drawing from the findings of the thematic analysis to highlight the different areas which were important to the community members.

At the beginning of the community engagement activities, the district leadership and community stakeholders thought COVID-19 was non-existent in Kalungu district. However, during the study launch all those attending were offered voluntary testing and over 30 SARS-CoV-2 positive cases were diagnosed just from the study launch out of about 100 people attending that meeting. Against this background, the Kalungu district leadership, health workers, community advisory board members and the village health team asked for their communities to be sensitised about COVID-19.

### The value of building on established relationships

The leadership at district, town councils, sub county and village local council levels welcomed the COVID-19 community surveillance study; partly because the organisation undertaking the surveillance (MRC/UVRI and LSHTM Unit) had been in the area for 30 years and was well-known:

*“Since the MRC has been in the community for so long, the study shall be accepted and it will be welcomed”*, Religious leaders Kyamulibwa.

This response was also partly because at the start of the pandemic there was considerable alarm over possible morbidity and mortality, and the local leadership wanted support in protecting the population.

The district, sub-county and town council leadership noted that the study was timely and further requested the MRC/UVRI and LSHTM Unit to continue to sensitise not only the proposed study communities but also all communities in Kalungu district. The leaders gave their support to the study:

*“We appreciate the work that the Unit has been doing in our community and we pledge that we will inform the community whenever we have a platform in any gatherings in the community about this COVID-19 study.”* Local leaders Kyamulibwa.

### Including everyone in the study

The leadership in Lukaya and Kyamulibwa town councils and Kyamulibwa sub-county, including the GPC community advisory board, expressed their dissatisfaction with the proposed study sample size selection methods of randomisation. They suggested inclusion of everyone or at least the local council leadership and the village health teams as opposed to random selection of only 500 households. They considered the study as a way to access testing and treatment for all, and wanted the benefits shared.

*“If you skip households, the next time you come up with any Unit programme or if you come to bleed, some may refuse and will tell you to bleed those you gave posho (compensation for study participation) last time (selected for COVID-19 community surveillance)”*, Local leaders Kyamulibwa.

*“Screening and testing for COVID-19 should be to all community members, both randomised and non-randomised. If this is not possible then at least consider giving priority to community/local council leaders”*, local council leaders and VHTs Lukaya town council.

In addition, they expressed their reservations about the monthly blood draws, nasal and throat swabbing because the procedures were painful. They asked whether there was a user-friendly way of testing for COVID-19. This was after some had had an opportunity to test for COVID-19 either at the study launch or at the study participating clinics.

At all meetings and engagement activities, the study team tried to explain the importance of random selection of study participants and how including everyone within the study area would not be feasible in terms of resources and time. In addition, the study team advised that in case anyone within the study area had symptoms suggestive of COVID-19, they could easily go to the three clinics and be tested.

The religious leaders across the different religions in Lukaya town council requested the study team to include mention of God in their COVID-19 community sensitisation messages to encourage participation:

*“You know when God does something and you don't listen, he may strike you again, so we shall tell people that God is sad”*, religious leaders in Lukaya.

Request to address other COVID-19 related needs

All the religious and local leaders, the police in Lukaya town council and village health teams in Kyamulibwa town council and sub-county requested the MRC/UVRI and LSHTM Unit to also work on other community needs like face masks and food (because of loss of income during the extended lock-downs). In addition, they also asked for treatment of those diagnosed with COVID-19.

*“Government promised masks to the communities but in Lukaya they have never been delivered so our people do not have masks and the Unit should help us give masks to people participating on study”*, Local council leader Lukaya town council.

Furthermore, health workers in the community engagement sessions suggested that the COVID-19 screening and testing services be extended to other health centres in Kalungu district other than Kyamulibwa and Lukaya health centre IIIs.

*“We understand you are providing COVID-19 screening services at the Kyamulibwa and Lukaya health centre III, but the COVID-19 problem is everywhere. Why don't you consider extending the service to [names of other health centres] health centre III's”*, Health workers attending the home based care training at Kyamulibwa field station.

In view of these requests, one reusable cloth face mask was provided to the enrolled participants. In addition to this, during the early study implementation, which coincided with the national wide lockdown, the study participants were compensated with maize flour and a bar of soap. However, when the lockdown was eased and as a result of feedback through community engagement, the study protocol was amended to monetary compensation.

### Provision of treatment for COVID-19

There was a need from the Kyamulibwa town council and sub-county local community leadership for the MRC/UVRI and LSHTM Unit to help provide treatment for those who were diagnsed with SARS-CoV-2 infection. This was mainly after the introduction of the Home-Based Care (HBC) programme in which people with mild COVID-19 symptoms were treated and cared for at home. There were fears of infected people passing on the infection to their household members and the community. The requested support for treatment was provided.

### Fears and stigma towards COVID-19 patients and affected families

There were fears and stigma towards COVID-19 patients and their families from the community, once they knew that a given member of a household was infected. This stigma went on even after people were no longer isolated, after completing 14 days of quarantine/isolation. The stigma did not stop at the infected individuals but extended to all the household members. A village health worker told us about a neighbour who shouted at a child from a family that had a SARS-CoV-2 positive person: *“Do not come here. You go back to your home because we are told you have COVID-19”*.

This fear was reminiscent of the days when many new HIV-cases were being identified in the 1990s, before treatment was available; this is not surprising since both were emerging infections which no one had experienced before. As the COVID-19 pandemic progressed and people recovered, the fear subsided.

### Misinformation on safety and efficacy of the COVID-19 vaccines

The COVID-19 pandemic and the vaccines were plagued by scepticism and social media misinformation which resulted in the low uptake of vaccines in Uganda. Vaccination started in March 2021 and by June 2021, only 770,000 Ugandans had received their first dose. There was considerable reluctance in the study communities to take up vaccination – with many people picking up information from social media and by word of mouth through which misgivings that the vaccines were not to be trusted were shared.

*“We hear that these vaccines are up to no good. They say that they may make us barren”*, Youth leader Kyamulibwa

COVID-19 vaccine hesitancy was not helped when the stakeholders heard that some European countries were refusing to use the vaccine type which had been offered to Uganda.

*“We hear from the media that the AstraZeneca vaccine has been rejected by the Europeans because it's dangerous to people's health”*, Local council leader Kyamulibwa.

Beliefs and understandings did change over time. At first people were reluctant to get vaccinated, and gave the misinformation they were seeing on social media as the reason for their reluctance. However, when people became aware of increased infections and deaths during the second wave, we saw a change in vaccination seeking behaviour which resulted in increased uptake.

Worries, fears and challenges of the Home Based Care (HBC) programme

At the start of the pandemic in Uganda in March 2020 up to peak of the first wave in November 2020, all COVID-19 cases were evacuated to designated government treatment and care centres. As the scarce medical resources were stretched to the limit, coupled with the realisation that up to 75% of cases were asymptomatic or had mild disease which did not need hospitalisation, a new policy of HBC was introduced. The patients who were recommended for HBC were those cases with either no symptoms or those having mild symptoms. However, because of the widespread fear of COVID-19 community neighbours did not want people with COVID-19 patients in homes close to theirs, and family members in the same houses were even more fearful because of the small space they shared.

*“If COVID-19 is a contagious disease, how are we going to live with people in the same home without getting infected. Moreover, most of our homes do not have many bedrooms”*, Randomised household member in a local council meeting Kyamulibwa.

### Protecting ‘frontline’ workers

As the COVID-19 community surveillance study progressed, SARS-CoV-2 vaccination was rolled out, starting with the essential workers, persons aged 50 and above and those with co-morbidities such as HIV, cancer, diabetes, hypertension and so forth. The essential workers, which included the health workers, teachers and security personnel were considered frontline workers with a higher risk of getting COVID-19. The vaccination exercise in Uganda started in March 2021. Not surprisingly, those involved in the supporting the surveillance study saw themselves as frontline workers too

*“As community leaders we are also regarded as frontline workers in our villages. We are responsible for mobilising and guiding the research teams in carrying out this research so we should be considered for vaccination like you people”*, Local council leader Kyamulibwa.

The MRC staff had no involvement in the vaccine rollout, beyond encouraging people to take up the offer of vaccination, and could not do anything to support this request, much to the disappointment of the community leaders.

Despite this disappointment, the community engagement activities revealed that the Kalungu district, community stakeholders and health workers at Kyamulibwa and Lukaya health centre IIIs appreciated that COVID-19 was a problem to local communities. This was contrary to the initial thinking expressed in the first month of the pandemic that COVID-19 was a problem in faraway communities.

## Discussion

The community engagement process/activities described in this paper built on the infrastructure that was already in place to support the GPC studies. Because of this history of involvement in research, many community members had experience of engagement in previous studies. The community advisory board with representation from the different community stakeholders promoted engagement and provided feedback on the research which informed the community engagement process^20^, ^24^.

Through our community engagement activities, we established that community, opinion, local council, religious leaders and health workers in Kalungu district were able to gain a clear understanding of COVID-19 as a new global public health problem at a time whn very little was known about it. Against this background, our community engagement activities resulted in calls for community sensitisation, problem-specific interventions to address COVID-19, such as face masks and urban centres' short food supply during lockdowns. Successful COVID-19 community surveillance called for a well-planned process and experienced personnel team to spear head quick data/sample collection.

Our findings are consistent with those from Tanzania in a study that used similar approaches to engage the community in COVID-19 prevention measures and gain a better understanding of the COVID-19 pandemic^25^. Elsewhere, community partners and members highlighted that in times of crisis and hardship, it was critical to initially come together to address immediate needs like basic health and social needs in equal measures over research^26^. A closely related community engagement approach that aimed at raising community COVID-19 awareness through virtual community halls witnessed development of medical-religious partnerships and strengthening of the University-community leadership partnerships. As in our community engagement efforts, the participants in the virtual community halls, asked the organizers to widen the coverage scope to include food and face masks distributions^18^, ^27^-^30^.

Our study has benefited from the existing local government and General Population Cohort infrastructures of clinical staff, district and community leadership, community advisory board, village health teams^20^, ^21^, ^24^. As Johnson and Goronga^31^ argued, lessons can be taken from the Ebola experience: community leadership must be involved in promoting the outbreak/pandemic response. Leveraging the existing community networks of religious leaders and village leadership, for example, in our study allowed quick information sharing and feedback collection^20^, ^24^. In other studies local community leadership has been engaged in preparation and implementation of the COVID-19 pandemic response^6^, ^32^, ^33^. In a seroprevalence study in a rural South Indian district village health committees cooperated to strategically position kiosk-based recruitment in the village and ensure the neighbourhood was able to take care of the affected household with food and essential medicines^34^. Community engagement depended on two-way communication which allowed actors to get involved in identifying issues, co-design interventions and responses. A study in Vietnam showed that successful community engagement was a dialogue with communities and stakeholders. Trust should be established through multiple channels with transparent, accurate and consistent information to address the rumours and misconceptions^6^, ^7^, ^35^.

## Study strengths and limitations

The study strengths included: the ability to put to use the existing research and human resources to put in place an effective community engagement process. The COVID-19 community surveillance study was carried out, in part, within the long-standing GPC). The second study area for the GPC COVID-19 community surveillance study was the Lukaya town council, an area that the Unit has carried out several studies before. Both study areas were located in Kalungu district where the leadership enjoy a good working relationship with the MRC/UVRI and LSHTM Unit. The other study strength lay in the ability to engage the community in the research process including developing participant recruitment strategies, consenting and time compensation.

A limitation of this study was the over reliance on the local and religious leaders together with the community advisory board. This is an inherent bias towards unique capabilities, time and spheres of influence of each and is unlikely to yield representation of all members of the communities. The community engagement approaches were designed to fit the study context, which may not be replicated elsewhere.

## Conclusion

Successful COVID-19 community surveillance calls for a two-way continuous active community engagement effort as opposed to one-time engagement between the research team, and the different stakeholders within the community. This should happen in an environment maximising on previous work gains, using existing resources and relationships as the basis of trusting community engagement when faced with new similar threats/emergencies.

However new a disease is, and however complex and frightening the screening procedures may seem, community engagement activities that involve community leadership and the participating households; coupled with the history of previous good service delivery over the years, will increase the chances of success for new projects being implemented in our community.
